# Long-Term Neuropsychiatric Sequelae in a Survivor of Cyanide Toxicity Patient With Arterialization

**DOI:** 10.7759/cureus.8430

**Published:** 2020-06-03

**Authors:** Rakan M Alqahtani, Mohammed Yousef Alyousef, Zaki Hassan AlWatban, Mohammed Khaled Ghandour

**Affiliations:** 1 Critical Care, College of Medicine, King Saud University, Riyadh, SAU

**Keywords:** case report, cyanide toxicity, long term neuropsychiatric sequelae

## Abstract

Cyanide is one of the most rapidly acting poisons and accounts for many suicidal and homicidal deaths. Some natural products such as silk and wool can release cyanide when burned. Most patients who survive cyanide poisoning experience neurological sequelae.

This report describes the case of a healthy 45-year-old Yemeni woman who was present during the burning of furniture in a closed space in her home. Upon admission, she displayed signs of inhalational injury, a black discoloration around her mouth and nostrils, and a first-degree burn on the left side of her neck. She experienced neuropsychiatric sequelae of cyanide poisoning, with deficits evolving over three months. Even after three months of treatment and continuous follow-up, she still showed signs of mild cognitive memory impairment along with word-finding difficulties and focal dystonia of her right hand.

Full neurological and cognitive assessments are crucial to determine the neuropsychiatric sequelae of acute cyanide toxicity. Magnetic resonance imaging (MRI) can show the extent and structure of lesions in cyanide-sensitive regions of the brain, but it is not always diagnostic. The arterialization of venous blood gases may serve as an early clue to the diagnosis of cyanide poisoning.

## Introduction

Cyanide is a lethal compound because it binds to ferric iron in cytochrome oxidase a3, thereby inhibiting oxidative phosphorylation, which leads to the depletion of intracellular adenosine triphosphate (ATP) [1]. Although some natural products such as silk and wool can release cyanide when burned, practically any substance with carbon and nitrogen can release cyanide when burned [2]. Cyanide toxicity should be suspected in smoke inhalation patients with two or more of the signs of neurological dysfunction, such as changes in mental status, loss of consciousness, and seizure activity [3]. These neurological aspects of cyanide poisoning can also be analyzed by structural neuroimaging [4,5]. Because the early clinical manifestations of cyanide intoxication are often nonspecific, emergency physicians must recognize the physiological abnormalities characteristic of cyanide poisoning. Assays of venous blood gases may aid in the diagnosis of cyanide poisoning [6]. This study describes the long-term neuropsychiatric sequelae in a survivor of cyanide toxicity due to inhalation, as assessed by arterialization. These patients have the potential for cognitive recovery.

## Case presentation

A healthy 45-year-old Yemeni woman was brought to the Accident and Emergency Department (ED) at King Saud University Medical City after being trapped in a closed space in her house for one hour while furniture was burning before the fire department and paramedics extracted her from the room. Upon admission, she showed signs of inhalational injury, a black discoloration around her mouth and nostrils, and a first-degree burn on the left side of her neck. She was found to have a Glasgow Coma Scale (GCS) score of 5. She was immediately intubated using a glideslope for airway protection. Measurement of vital signs showed a blood pressure of 90/65 mmHg, a respiratory rate of 18 breaths per minute, and a heart rate of 102 beats per minute. Resuscitation with intravenous fluids was initiated. Venous blood gas showed a pH of 7.211, PO2 of 79.4 mmHg, O2 saturation of 95%, PCO2 of 35.6 mmHg, and HCO3 of 15 mEq/L.

**Figure 1:**
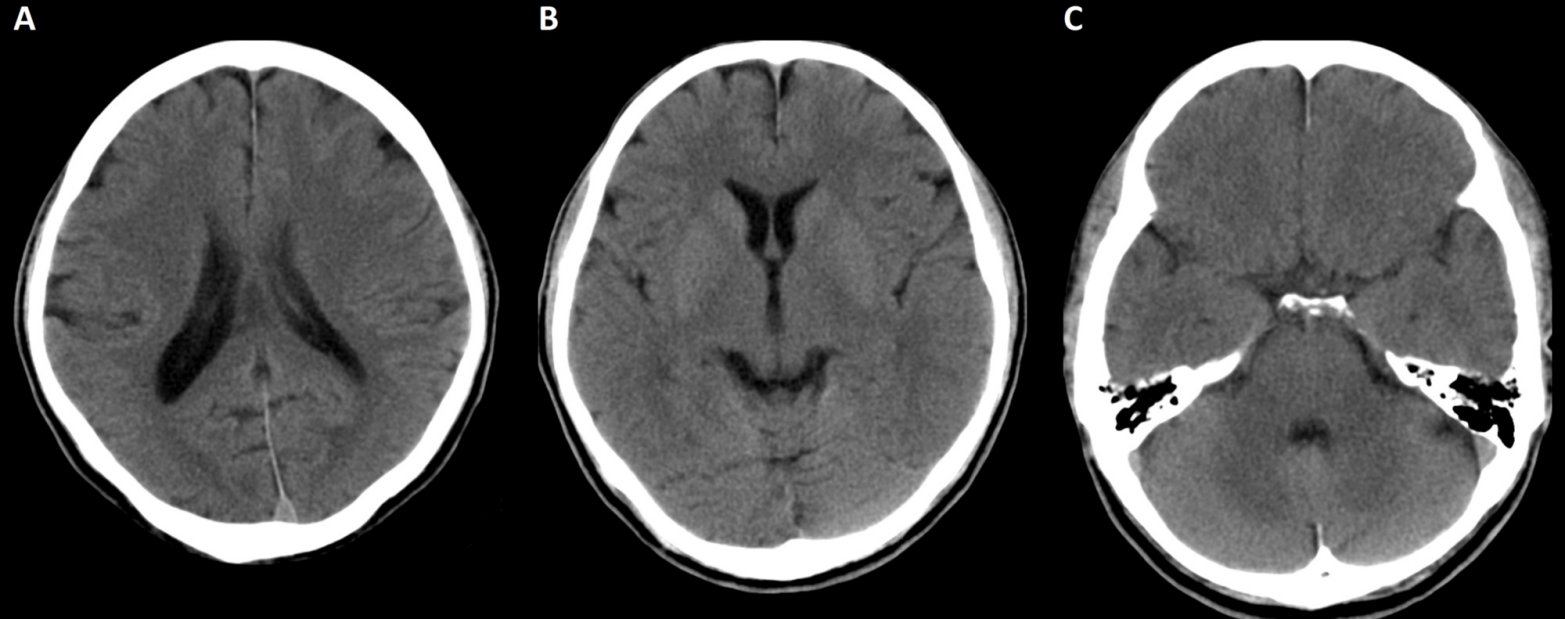
CT of the brain on admission. There is no evidence of acute insult or cerebral edema on the CT after the administration of the Lilly Cyanide Antidote Kit. (A) CT of the brain at the level of the body of the lateral ventricles: frontal, parietal, and occipital lobes are not affected. (B) CT of the brain at the level of basal ganglia: normal caudate nuclei, globi pallidi, and putamina. (C) CT of the brain at the level of cerebellar hemispheres: no evidence of ischemic insult at the temporal and cerebellar hemispheres.

**Figure 2:**
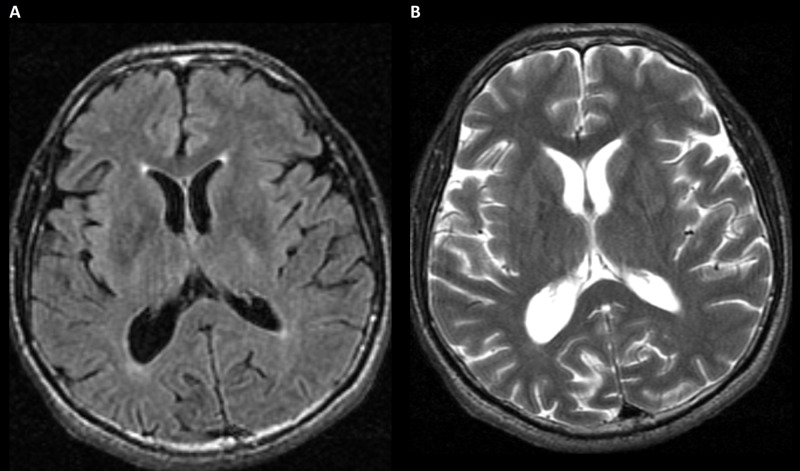
MRI upon admission. (A) FLAIR image. (B) Axial T2 image. There are no hyperintensity signals in the basal ganglia or hippocampal regions. FLAIR, fluid-attenuated inversion recovery

Because acute cyanide poisoning was suspected, she was administered the contents of one Lilly Cyanide Antidote Kit (Eli Lilly and Co., Indianapolis, IN, USA), containing amyl nitrite, sodium nitrite, and sodium thiosulfate, two hours after arrival at the ED. The patient was transferred to the intensive care unit (ICU), where she received controlled mechanical ventilation with high sedation to overcome asynchrony, along with multiple sessions of chest physiotherapy and nebulization treatment as well multiple evaluations by bronchoscopy. Despite improvements in her overall clinical status, including hemodynamics, oxygenation, and acid-base imbalance, her GCS improved only slightly. Brain computed tomography (CT) scan and magnetic resonance imaging (MRI) were unremarkable, with no indications of anoxic brain injury or acute brain insult (Figures 1, 2). On the fourth day, her GCS was 13 to 14, and she was extubated despite signs of delirium. These signs improved the next day, and she was transferred to the ward. She developed hospital-acquired pneumonia two days later and was started on treatment with piperacillin-tazobactam and vancomycin. She later developed stage II acute kidney injury (AKI), as shown by an increase in serum creatinine concentration from 105 ng/dL to 210 ng/dL. By day 15, she had improved clinically, but her speech was slurred, her hand movements were abnormal, and her sleep patterns were disrupted. She also showed a loss of short memory and experienced flashbacks of the incident.

The neurological evaluation showed focal dystonia in her right hand, short-term memory loss on the Montreal cognitive assessment test, and word-finding difficulty. There was no evidence of gait instability, parkinsonian features, sensory or motor deficits, or cerebellar signs.

**Figure 3:**
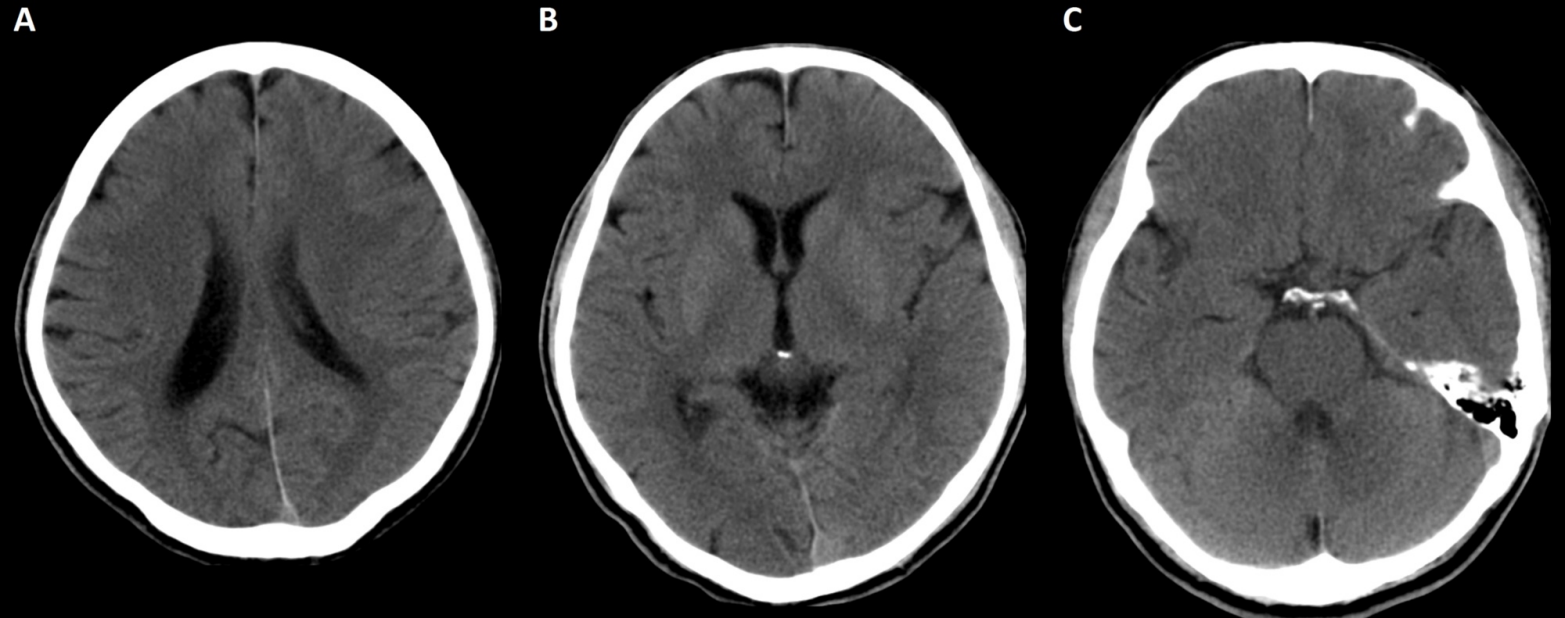
A three-month follow-up CT of the brain. There is no evidence of structural damage after three months despite the focal dystonia and mild cognitive impairment. (A) The body of the lateral ventricles cut with no evidence of chronic structural damages on the frontal, parietal, and occipital lobes. (B) The basal ganglia (the most common site for structural brain insult after cyanide toxicity) are not affected and the patient did not show any features of extrapyramidal signs. (C) No evidence of chronic ischemic changes was noted on temporal and cerebellar areas.

**Figure 4:**
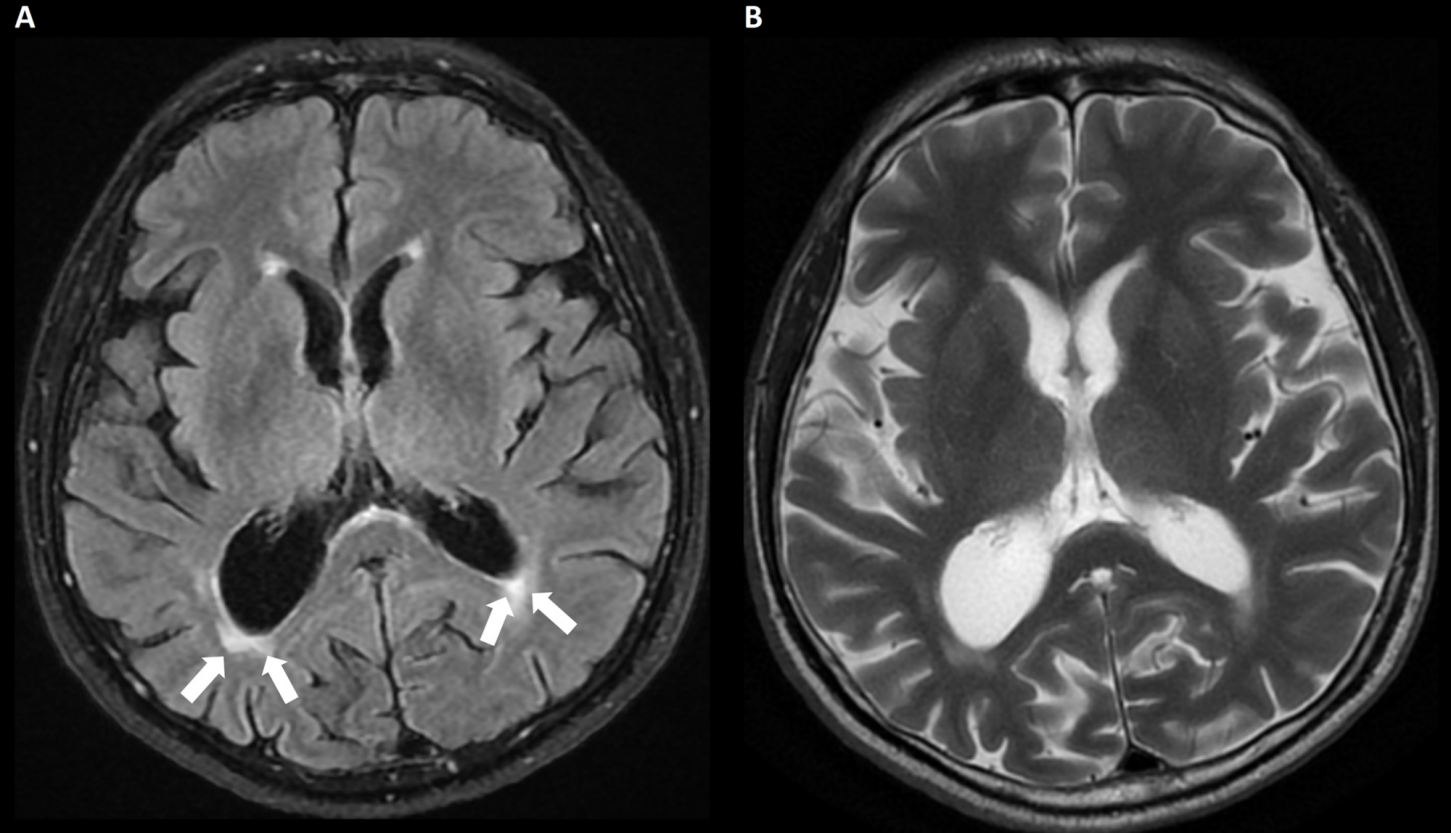
A three-month follow-up MRI. (A) FLAIR image. (B) Axial T2 image. Non-specific mild periventricular hyperintensities on FLAIR image (arrows) can be seen, with otherwise unremarkable features at the basal ganglia or hippocampal regions. FLAIR, fluid-attenuated inversion recovery

A three-month follow-up CT scan was negative, and a follow-up MRI showed increased hyperintensity signals in the periventricular regions but was otherwise unremarkable. Electromyography and cerebrospinal fluid analysis were unremarkable. The psychiatric examination indicated mild depression, with no major changes in mood or behavior. It was too soon after the incident to assess for post-traumatic stress disorder. Three months later, she still had mild cognitive memory deficit along with word-finding difficulties and focal dystonia of her right hand (Figures 3, 4).

## Discussion

Cyanide toxicity is due to its binding to ferric ion in the a-a3 complex of cytochrome oxidase in the mitochondrial respiratory chain. This binding blocks oxidative phosphorylation catalyzed by cytochrome oxidase, thereby inhibiting aerobic metabolism, which rapidly leads to depletion of ATP, resulting in cell injury or death [7].

Many sources of cyanide exposure have been identified, including smoke, nitriles, agriculture, mining, amygdalin, and hydrogen cyanide gas [8]. The clinical manifestations of cyanide toxicity depend on the dosage, route, duration, and source of exposure. Cyanide can be absorbed by the body in multiple ways, such as oral, dermal, parental, and inhalational routes [9]. Cyanide toxicity in our patient was due to her one-hour-long inhalation of smoke resulting from the burning of furniture in a closed space in her home.

Initial manifestations of cyanide toxicity include nausea, vomiting, headache, tachypnea, flushing, and dizziness, but none of these is specific to cyanide toxicity [10]. The most frequently reported signs of cyanide toxicity are unresponsiveness, which is observed in 78% of patients, followed by respiratory failure (73%), arrhythmia (72%), hypotension (54%), cardiac arrest (20%), seizures (20%), cyanosis (15%), almond odor (15%), and cherry-red skin (11%). These signs can help narrow the differential diagnosis [9].

The arterialization of venous blood gases may serve as an early clue to the diagnosis and severity of cyanide poisoning. Venous oxygen saturation is determined by oxygen uptake, arterial oxygen concentration, hemoglobin concentration, and cardiac output. If other factors remain constant, inhibition of peripheral oxygen uptake by cyanide will elevate venous oxygen saturation [11].

Cyanide poisoning with significant arterialization tends to bilaterally affect the basal ganglia, especially the striatum, and the cerebral cortex, especially the sensorimotor cortex [12]. All of these common findings are not evident in our case. More severe cyanide poisoning can lead to more widespread changes, including diffuse cerebral edema, classically seen as bilateral hypo-attenuation in the basal ganglia and reflective of necrosis [13]. These areas may or may not show evidence of hyper-attenuating macroscopic hemorrhage [14]. Cortical variations are often not evident on CT [14]. The same regions of involvement are detected by CT and MRI, but the latter provides more detailed information. Electrocardiogram findings of cyanide toxicity include asystole, atrial fibrillation, tachycardia, bradycardia, and ventricular arrhythmias, making electrocardiography diagnostic in patients with cyanide toxicity [9].

Cyanide toxicity can be easily treated if detected early. Treatments of cyanide toxicity include supportive care with supplemental oxygen (79% of patients) and intravenous fluids (66%). Many patients develop respiratory failure and must undergo intubation (66%). Also, some patients develop refractory hypotension requiring vasopressor support (39%). Overall, 20% of patients are administered cyanide antidote kits (sodium nitrite, amyl nitrite, and sodium thiosulfate), 29% receive hydroxocobalamin, 40% receive sodium thiosulfate alone, and some receive multiple antidotes [9].

Hospital-acquired pneumonia, defined as the development of pneumonia ≥ 48 hours after hospital admission, is the second most frequent type of nosocomial infection in ICU patients and the leading cause of death of patients with hospital-acquired infections [15]. Unfortunately, our patient developed hospital-acquired pneumonia two days after admission and was started on treatment with piperacillin-tazobactam and vancomycin. She subsequently developed stage II AKI, as evidenced by elevated creatinine concentration.

Because early treatment is critical in patients with cyanide toxicity, early diagnosis is essential. Survivors of severe cyanide poisoning may experience subsequent complications including Parkinson's disease and other types of neurological sequelae. The basal ganglia are sensitive to cyanide toxicity. Chronic cyanide exposure can lead to non-specific symptoms including headache, abnormal taste, vomiting, chest pain, and anxiety [16]. Our patient did not experience gait instability, parkinsonian features, sensory or motor deficits, and/or cerebellar signs since she received the antidote as soon as she arrived at the ED.

## Conclusions

The time factor in cyanide antidote administration is often overlooked in the clinical settings. The administration of antidote within a two-hour period may help ameliorating the neurological complications resulting from acute cyanide toxicity with venous arterialization. Establishing early diagnosis with adequate history-taking and using the arterialization of the venous blood gas as a red flag should be the main priorities in approaching any suspected case in ED. Long-term mild neuropsychiatric sequelae such as mild cognitive impairment and focal dystonia may ensue and may necessitate neurological and psychiatric clinical evaluation as a guide for further management as opposed to the radiological surveillance.
